# Olaparib treatment in older patients with ovarian cancer: need for ‘real-world’ data beyond clinical trials

**DOI:** 10.3332/ecancer.2020.1104

**Published:** 2020-09-15

**Authors:** Gabor Liposits, Christian Nielsen Wulff, Anne Otland, Lars Ulrik Fokdal

**Affiliations:** 1 Department of Oncology, Region Hospital West Jutland, Gl. Landevej 61, Herning 7400, Denmark; 2 Department of Oncology, Aarhus University Hospital, Palle Juul-Jensens Boulevard 99, Indgang D3, Plan 2, Krydspunkt D203, 8200 Aarhus N, Denmark; a https://orcid.org/0000-0002-8204-3949

**Keywords:** olaparib, older patients, geriatric assessment, ovarian cancer, real-world data, quality of life

## Abstract

**Background:**

Ageing is a risk factor for cancer. Worldwide, the number and proportion of adults aged ≥65 will increase, along with the incidence of ovarian cancer. Older adults are under-represented in randomised clinical trials (RCTs), and those who are enrolled have a good performance status and no major health issues. These patients are not representative of older patients seen in everyday clinical practice; therefore, age-specific data on efficacy and toxicity of olaparib in the ‘real-world’ setting are lacking.

**Methods:**

This observational study was conducted in the Central Jutland Region in Denmark. Data in unselected older (≥65) patients with known BRCA mutation receiving olaparib maintenance treatment for platinum-sensitive relapsed ovarian cancer were registered between 2015 and 2019. Toxicity and progression-free survival (PFS) were registered. No geriatric assessment has been performed.

**Results:**

In total, 20 consecutive patients ≥65 years were included with a median age of 75 years (range: 65–85). Most of the patients (18/20) had ECOG PS: 0–1. Treatment interruption and dose reduction occurred in 65% of the patients. Toxicities of any grade occurred in 18 (90%), whereas grade 3/4 toxicities occurred in 6 patients (30%). Treatment was terminated due to disease progression or unacceptable toxicity in 13 (65%) patients. The median PFS was 6 months (range: 2–31), and the median follow-up was 15 months (range: 3–30).

**Discussion:**

Our ‘real-world’ experience shows that unselected older patients represent a significant larger proportion in real life than in RCTs; furthermore, older patients in a real-world setting may experience more side effects possibly affecting the quality of life. The median PFS data suggest that older patients may not derive the same clinical benefit than their fit and younger counterparts.

There is a need to enrol vulnerable/frail older patients into RCTs, ensuring that data will also be applicable in standard clinical settings. Incorporating geriatric assessment into these trials should be encouraged.

## Background

Ageing is a risk factor for cancer [1]. Due to the ageing of the population, the percentage of the European population aged over 65 years is projected to increase from 17.1% in 2008 to 30.0% in 2060 [2]. In parallel with the demographic shift of the population, the incidence of ovarian cancer is expected to rise [3, 4].

In 2012, a total of 565 patients were diagnosed with ovarian cancer in Denmark, and 41.9% were 70 years or older. The incidence rates among older patients were three times, and the mortality rates were 3–4 times higher than in patients aged <70. Well known that the outcomes in older women with ovarian cancer are worse, and the probability of receiving standard treatment, in accordance with guidelines, is reduced by 50% [6, 7].

The randomised controlled trials (RCTs) are the gold standard guiding the management of patients with cancer. Older adults are frequently under-represented in RCTs [6–10], and those who are enrolled are typically in good performance status (ECOG 0-1) and have adequate organ functions [11]. A pooled analysis of eight prospective phase I/II trials [11] including patients (*n* = 398) with recurrent ovarian cancer who received olaparib capsules showed that 19.6% (*n* = 78) of patients were 65, and only 10% (*n* = 40) were 70 years or older. All patients received the maximum tolerable dose of olaparib (400 mg bid), suggesting that these patients were fit and had no significantly impaired organ functions. In general, these fit older patients do not represent the majority of older patients seen in everyday clinical practice, who are often vulnerable and have comorbidities (Figure 1) [6].

A currently published review by the Young International Society of Geriatric Oncology [12] concluded that age-specific data in older patients with ovarian cancer, treated with poly ADP-ribose polymerase inhibitor (PARPi), are lacking. Hence, sparse available evidence suggests that fit older patients with ovarian cancer may benefit from PARP inhibitors in the maintenance setting [13–17]. The health-related quality-of-life (HRQoL) data regarding older patients with ovarian cancer treated with olaparib are completely lacking.

## Methods

This observational study was conducted in the Central Region of Jutland, Denmark, in the two departments of oncology which treat patients with ovarian cancer (Department of Oncology, Regional Hospital West Jutland, and Department of Oncology, Aarhus University Hospital). ‘Real-world’ data in unselected older (≥65) patients with known BRCA mutation (germline and/or somatic) receiving PARPi maintenance treatment for platinum-sensitive relapsed ovarian cancer starting treatment between 2015 and 2019 were retrospectively collected. The maintenance of PARPi treatment was given in the form of olaparib capsules, starting dose 400 mg twice daily in 4-week cycles either until progression or unacceptable toxicity. Toxicity and progression-free survival were registered. No comprehensive geriatric assessment has been performed; however, some patients received geriatric screening.

## Results

In total, 20 consecutive patients (Table 1) aged ≥65 years were included with a median age of 75 years (range: 65–85). All patients received at least one previous line platinum-containing chemotherapy for relapsed disease. Eighteen patients had ECOG PS: 0–1, whereas two patients had ECOG PS: 2. Treatment was terminated due to disease progression or unacceptable toxicity in 13 (65%) patients. The median progression-free survival (mPFS) was 6 months (range: 2–31), and the median follow-up was 15 months (3–30). Treatment interruption and dose reduction occurred in 13 patients (65%). Toxicities of any grade (Table 1) occurred in 18 patients (90%). Most common side effects were fatigue (70%), nausea/vomiting (60%) and anaemia (20%). Grade 3/4 toxicities occurred in 6 patients (30%), and anaemia, infection, nausea/vomiting, fatigue and neutropenic fever were registered, respectively. Two patients experienced a significant deterioration in physical function and mental well-being due to recurrent infections, fatigue and depression, respectively. After olaparib treatment was stopped, improvement in physical function and mental well-being was observed.

## Discussion

Although our observations have several limitations, these data should be put in context getting the right interpretation. The number of included patients (*n* = 20) seems modest although, to the best of authors’ knowledge, the cohort is the largest cohort of consecutive unselected older patients received olaparib treatment.

In this region, in total, 39 patients received olaparib maintenance treatment, of whom 20 (52%) were 65 years or older, whereas, in the largest published cohort, which was a pooled analysis of eight trials [11], only 19.8% suggest that unselected older patients represent a significant larger proportion in real life than in RCTs, because of the selection bias in phase II/III RCTs including exclusively fit older patients.

The toxicity profile of PARPi appears to be acceptable based on available data from RCTs although the occurrence of clinically significant toxicity is likely to be higher in ‘real-world’ settings. The data show that older patients in real-world setting experience more side effects possibly affecting HRQoL negatively. A possible explanation could be that high-grade peak toxicity may not reflect the full toxicity burden of PARPi. Chronic low-grade toxicities may be even more important affecting HRQoL for this group of patients [19]. In addition, recently published papers [18, 20] elucidated that publications reporting the effectiveness and safety of cancer drugs often use subjective terms that downplay the seriousness of adverse events. A representative example is the SOLO2 trial, where olaparib did not improve the prespecified primary quality-of-life analysis, and this was interpreted as ‘olaparib is not having a significant detrimental effect on QoL’ and ‘there were clinically meaningful patient-centred benefits despite the adverse effects’ [18].

The mPFS was shorter in this cohort than mPFS data in phase II trial (6.0 versus 11.2 months) based on olaparib received approval, suggesting that older patients may not derive the same clinical benefit than their fit and younger counterparts. Thought provoking that olaparib received accelerated approval based on response rates alone and was required to assess PFS and OS in confirmatory trails. However, olaparib received full approval based on improved PFS alone. At the time of full approval, overall survival data were not yet matured [20]. Given the high cost of treatment with PARPi and the economical strain on the healthcare systems worldwide, it is essential to identify the older patients who are likely to benefit from PARPi. Omitting the treatment of older and vulnerable patients who do not benefit of PARPi treatment means avoidance of toxicities, hospitalisations and less healthcare expenses.

The above-mentioned data suggest that there is a need to enrol vulnerable or frail older patients into RCTs to secure that results from RCTs will also be applicable in a standard clinical setting. In addition, incorporating geriatric assessment into these trials should be encouraged to adequately risk-stratify older patients [21–27] and to help balance the potential benefits and harms of PARPi treatment. The geriatric assessment may guide appropriate patient selection, avoid both over and undertreatment and, thus, may help preserve physical function and mental well-being while reducing the socioeconomic burden of this expensive cancer treatment.

## Conclusion

Older vulnerable patients receiving olaparib in a ‘real-world’ clinical setting experience more side effects possibly affecting quality of life negatively and have shorter mPFS benefit. RCTs including older unselected patients must ensure that data from RCTs will be applicable in the everyday clinical setting. Geriatric assessment should be an essential component of these trials.

## Future perspective

The working group is planning to start a prospective observational study including older patients with ovarian cancer treated with PARPi, where geriatric assessment-guided patient stratification is a central element, and the study will focus on mPFS, HRQoL and geriatric endpoints.

## Conflicts of interest

None.

## Funding statement

No funding was received for this work.

## Figures and Tables

**Table 1.** Detailed patient characteristics and treatment outcomes.

Patient	Age	ECOG PS & Comorbidity	Number of previous lines of platinum for recurrent disease	Response to last line platinum (Imaging and/or CA125)	Number of olaparib cycles & best response (Imaging and/or CA125)	Grade 3-4 toxicities	Grade 1-2 toxicities	Reason for discontinuation	Dose level
1	67	**1**—none	6	PR	0—permanent discontinuation due to allergic reaction	none	Gr. 2 urticaria due allergic reaction	Toxicity	0
2	68	**1**—Asthma, atrial fibrillation, short bowel-syndrome	1	PR	2—PD	none	none	Progressive disease	0
3	85	**0**—asthma, arthrosis, dyspepsia	1	PR	2—SD	**Gr. 3 nausea**	Gr. 2 fatigue, gr. 2 depression	Toxicity	−2
4	65	**1**—none	1	PR	3—SD	**Gr. 3 anemia**	Gr. 2 nausea, gr. 2 dizziness	Toxicity	−1
5	77	**0**—Atrial fibrillation, dyslipidemia, angina pectoris, arthrosis	1	PR	3—SD	**Gr. 3 infection**	Gr. 2 fatigue, gr. 1 anemia	Toxicity	0
6	79	**1**—surgery and adjuvant chemotherapy for colorectal cancer (2013), deep venous thrombosis and lung embolism	1	PR	3—SD	**Gr. 3 fatigue**	Gr. 2 nausea, gr. 2 diarrhea, gr.2 weight loss, gr. 2 anemia	Toxicity	−2
7	66	**1**—Asthma	1	PR	4—PD	none	Gr. 2 nausea, gr. 1 fatigue, gr. 1 neutropenia	Progressive disease	−2
8	75	**1**—none	2	PR	4—PD	none	Gr. 1 fatigue	Progressive disease	0
9	77	**1**—none	1	PR	4—PD	**Gr. 3 febrile neutropenia**	none	Progressive disease	−1
10	66	**2**—heart failure, hypothyreosis	1	PR	6—SD	none	Gr. nausea, gr. 1 fatigue	Progressive disease	−2
11	72	**2**—Chronic obstructive pulmonary disease, osteoporosis, sarcopenia, hypertension, depression, early breast cancer—received adj. chemotherapy, radiotherapy, aromatase inhibitor and trastuzumab (2016).	1	PR	7—SD	none	Gr. 2 nausea, gr. 1 fatigue	Progressive disease	−2
12	70	0—none	1	PR	8—SD	none	Gr. 1 fatigue, gr. 1 myalgia, gr. 1 abdominal pain	Progressive disease	0
13	73	**0**—dyslipidemia, osteoporosis	2	PR	9—SD	none	Gr. 1 fatigue, gr. 1 nausea, gr. 1 neutropenia	Ongoing	−1
14	71	**0**—osteoporosis, transient ischemic attack, lung embolism	4	PR	9—SD	none	Gr. 1 headache, gr. 1 fatigue, gr. 1 dyspnea gr. 1 blurred vision	Ongoing	0
15	67	**1**—breast cancer (2000)	1	PR	12—SD	none	Gr. 1 abdominal pain, gr. 1 nausea, gr.1 fatigue	Ongoing	0
16	77	**1**—hypertension	2	CR	14—SD	none	Gr. 1 anemia, gr. 1 fatigue, gr. 1 nausea	Ongoing	−2
17	78	**1**—pulmonary embolism, lung cancer (St.I) accidental fund—removed by surgery	2	CR	15—SD	none	Gr. 2 nausea, gr. 2 fatigue, gr. 1 elevated se- creatinine	Ongoing	−2
18	74	**0**—hypertension	1	CR	19—SD	**Gr. 3 anemia**	Gr. 1 nausea	Ongoing	−2
19	80	**1**—early breast cancer (1991)	1	CR	29—SD	none	Gr. 1 fatigue, gr. 1 diarrhea, gr. 1 nausea, gr. 1 dizziness, gr. 1 alopecia, gr. 2 neutropenia	Progressive disease	−2
20	77	**1**—hypertension, early breast cancer (2006)	1	CR	31—SD	none	Gr. 2 nausea, gr. 2 fatigue, gr. 1 elevated se- creatinine	Ongoing	−2
